# Cystic Fibrosis and CFTR Modulators: The Impact on Bone Density, Muscle Mass and Strength in Children and Young Adolescents

**DOI:** 10.3390/children12111434

**Published:** 2025-10-23

**Authors:** Katerina Iordanidou, Nikolaos D. Karakousis, Elpis Hatziagorou, Elisavet-Anna Chrysochoou, Maria Galogavrou, Athina Sopiadou, Maria Papagianni

**Affiliations:** 1 Department of Nutrition and Dietetics, School of Physical Education, Sport Science and Dietetics, University of Thessaly, 42100 Trikala, Greece; 2 Pediatric Pulmonology and Cystic Fibrosis Unit, Hippokration Hospital of Thessaloniki, Aristotle University of Thessaloniki, 54124 Thessaloniki, Greece; hatziagorou@auth.gr (E.H.); asopi@auth.gr (A.S.)

**Keywords:** cystic fibrosis, muscle mass, bone density, dual energy X-ray absorptiometry, ivacaftor

## Abstract

**Background/Objectives**: Cystic fibrosis (CF) is a multisystemic and genetic disorder. Mutations in the CF transmembrane conductance regulator (CFTR) gene impair the function of the CFTR protein, leading to various complications in multiple organs, mainly the lungs. **Methods**: In this article, we have tried to investigate the potential impact of CFTR modulators on bone density, muscle mass, and strength in children and young adolescents with CF by using the existing literature and conducting a narrative (non-systematic) review. **Results**: It has been demonstrated that CFTR modulators may positively influence bone mineral density. On the other hand, the impact of CFTR modulators on muscle mass and strength seems to vary among studies. **Conclusions**: Besides the current literature, further studies are needed to validate the existing claims.

## 1. Introduction

Cystic fibrosis (CF) is an autosomal recessive genetic disorder that affects multiple organ systems [1]. It is the most frequent genetic disorder in Caucasian subjects, with an occurrence of 1/2500–3500 live births, affecting approximately 50,000 people in Europe and over 85,000 globally [1,2,3]. Regarding this disease’s genetic profile and pathophysiology, a mutation in the CF transmembrane conductance regulator (CFTR) gene alters the CFTR protein, which controls the function of sodium and chloride channels at the cell surface epithelium [2,4]. The CFTR protein’s primary role is to allow chloride transport through the mucus-producing cells in the airway epithelium, followed by water, leading to a thinner mucus texture [2]. However, in CF, the defective CFTR protein results in the production of thick, sticky mucus, which can lead to the obstruction of the airways in the lungs and pancreatic ducts [2,5]. Among the affected organs, the lungs are the most severely damaged, leading to death in 90% of subjects living with CF [2]. In addition, CFTR is expressed in vascular smooth muscle cells and plays a role in vascular tone regulation, whilst its deficiency has been associated with skeletal muscle weakness and epithelial cell senescence [6,7].

At the time of CF initial description in 1938, life expectancy was short [8]. It is recorded that the median survival age increased from 29.0 years in 1990 to 38.6 years in 2012, prior to the introduction of CFTR modulators, and to 68.0 years in 2023, showing substantial improvement following the utilization of CFTR modulators [9]. With the advances in the care of patients with CF and the increase in their life expectancy, new clinical manifestations of the disease have emerged, including CF-associated bone disease [10]. In adults, the prevalence of osteoporosis and osteopenia is reported to reach 23.5% and 38%, respectively, while vertebral and non-vertebral fractures occur in 14% and 19.7% of cases, respectively [11]. There are also studies showing reduced bone density during childhood and adolescence [12,13], as well as others showing a decrease in bone density during the transition from childhood to adolescence [14,15]. A recent review has also shown that individuals with CF may have an increased risk of developing sarcopenia and osteopenia [16]. DXA is currently the gold standard for evaluation of CF bone disease and guiding therapy for osteoporosis in subjects living with CF, while other means might include high-resolution peripheral quantitative computed tomography (HR-pQCT) [17,18].

Factors that contribute to the CF-associated bone disease include malnutrition, sedentary lifestyle, endocrine disorders, pancreatic insufficiency, delayed puberty, vitamin D and K deficiencies, calcium malabsorption, and/or the use of exogenous glucocorticoids [19]. However, it appears that the degree of residual CFTR protein function can also affect, either directly or indirectly, the pathogenesis and progression of cystic fibrosis–associated bone disease. It has been suggested that osteoblasts express CFTR mRNA and protein, and that inhibition of CFTR-mediated chloride channel activity influences the release of osteoprotegerin and prostaglandin E2, both key regulators of bone formation [20]. Another study on mice has also indicated that the F508del-CFTR mutation may play a role in bone disease by impairing the rate of new bone formation in infants and young children with cystic fibrosis [21].

Since the discovery of the CFTR gene in 1989, more than 2000 mutations have been described to date [22]. The most common mutation is in the F508del allele, accounting for approximately two-thirds of CF cases globally [23]. Understanding the CFTR mutations that lead to the defective CFTR protein enabled the development of targeted pharmacological therapies [24]. Two types of CFTR modulators (potentiators and correctors) are now available for the treatment of patients with CF [25]. Ivacaftor is the only CFTR potentiator approved initially by the FDA for patients with the G551D mutation. Ivacaftor enhances the CFTR protein activity by increasing the frequency of CFTR channel opening on the cell surface [24]. Lumacaftor, tezacaftor, and elexacaftor are the currently approved CFTR correctors, and their role is to target directly the defective CFTR protein [26]. In 2019, the FDA approved a triple combination therapy consisting of two CFTR correctors, elexacaftor (ELX), tezacaftor (TEZ), and along with a potentiator, ivacaftor (IVA), ETI [24].

So far, many studies have shown that ivacaftor, either as monotherapy or in combination with the CFTR correctors, can improve the lung function and the quality of life of the CF patients [24,27,28,29,30]. However, less information is available regarding the impact of CFTR modulators on bone health. Therefore, the aim of this study is to review the current literature on the effects of CFTR modulators on bone mineral density (BMD), muscle mass, and strength.

## 2. Materials and Methods

**Figure 1:**
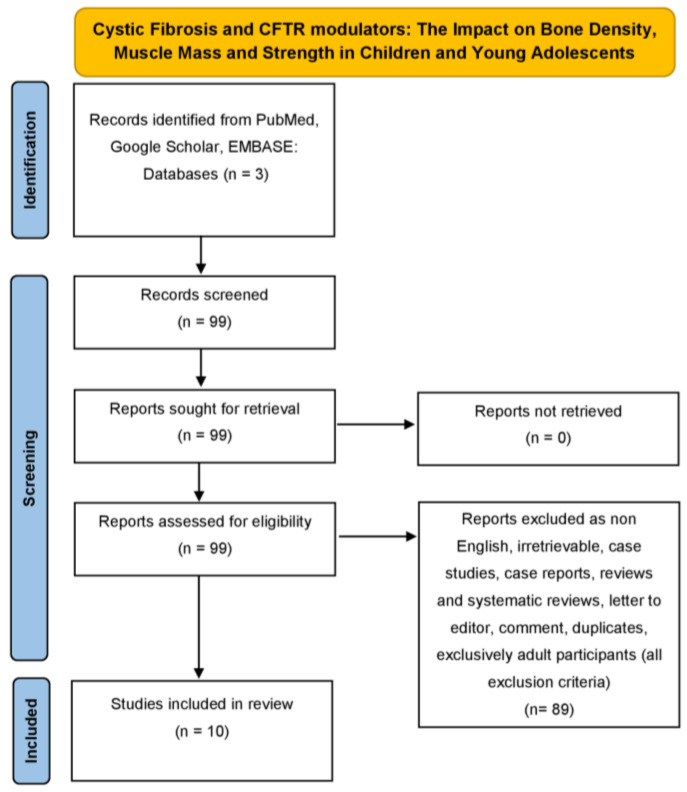
Narrative review search flow diagram (The PRISMA 2020 statement) demonstrating the literature review strategy related to cystic fibrosis and CFTR modulators interplay (only original, English, concerning children and young adolescents and non-animal articles were included in this review study).

We thoroughly examined the databases of PubMed, Google Scholar and EMBASE from November 2016, where the first article concerning our research was recorded, to September 2025, using the following combinations of specific keywords: “cystic fibrosis” and “dual energy X-ray absorptiometry” or “bone density” or “bone mineral density” or “muscle mass” or “muscle strength” or “sarcopenia” or “osteoporosis” or “osteopenia” and “ivacaftor” or “tezacaftor’ or “ elexacaftor” or “lumacaftor” or “deutivacaftor” or “vanzacaftor” to conduct research for a narrative non-systematic review article. Original studies written in English, concerning children and young adolescent population under investigation, were included in this review study. In addition, all the references related to the studies included were exhaustively examined. Studies concerning purely adult participants and animals were excluded. Duplicates were identified and removed manually. Our search strategy is depicted in the narrative review search flow diagram (The PRISMA 2020 statement) (Figure 1).

We conducted a narrative review study with a primary scope to provide an overview of the literature about a subject that has not been reviewed so far. No formal quality assessment tools were applied, as priority was given to summarizing the breadth of available evidence and highlighting emerging themes. While the absence of a structured appraisal is acknowledged as a limitation, it is consistent with the narrative review approach and purpose of this study, and as a narrative review of previously published studies, no ethical approval was required. 

## 3. Results

### 3.1. Database Search and Study Characteristics

An electronic literature review was performed on Pubmed, Google Scholar, and EMBASE from November 2016 to September 2025, and a total of 99 articles were retrieved. These 99 articles were assessed for eligibility. After applying the eligibility and exclusion criteria, a total of 89 articles were excluded. Finally, a total of 10 studies met our inclusion criteria for the outcomes, being bone density, muscle mass, and strength. Figure 1 presents the details of our search strategy.

Four of the included studies were observational studies, one was cross-sectional, one was a pilot study, three were prospective, and one was multicenter. The study characteristics are summarized in Table 1 and Table 2.

### 3.2. The Impact of CFTR Modulators on Bone Density in Children and Young Adolescents with CF

The impact of CFTR modulators on bone density was examined in four studies: an observational study, a pilot study, a cross-sectional study, and a prospective single-center study. Table 1 summarizes the main characteristics of the included studies.

Putman MS et al. investigated the impact of treatment with ivacaftor on bone health in children and adults with CF by conducting a prospective observational cohort study in a clinical setting over 2 years [31]. Their findings indicated that ivacaftor treatment improved cortical bone microarchitecture at both the radius and tibia in adults with cystic fibrosis, regardless of changes in lung function or body mass index [31]. However, in both pediatric and adult populations, baseline and follow-up dual-energy X-ray Absorptiometry (DXA) and high-resolution Peripheral Quantitative Computed Tomography (HR-pQCT) measurements showed no significant differences regarding the areal bone density among the cohorts [31].

A pilot prospective-retrospective study conducted by Gur M et al. reviewed the effect of Elexacaftor/Tezacaftor/Ivacaftor (ETI) on bone mineral density in young adults with CF (18.6 ± 4.7 years) 3 months after the initiation of the treatment, and they compared these results with the ones obtained 2 years ago [32]. A significant increase in hip and spine bone mineral density was recorded in the study group (0.73 ± 0.098 to 0.81 ± 0.12 gr/cm^2^ hip, *p* = 0.017; 0.76 ± 0.14 to 0.82 ± 0.14 gr/cm^2^ spine, *p* = 0.025), whereas the control group maintained stable BMD levels [32].

A prospective single-center study by Boni A et al. investigated growth patterns based on height velocity (HV) and assessed alterations in bone mineral density and body composition according to the CFTR variant genotype [33]. The study included 24 children (mean age was 8.7 ± 1.9 years), who were eligible for ETI treatment. Bone mineral density (BMD) was assessed using DXA scan, and body composition was assessed by bioelectrical impedance analysis (BIA). In conclusion, no significant differences were observed among genetic groups with respect to baseline BMD or lean mass [33].

Finally, Clayton LJ et al. in their cross-sectional study, compared 15 patients with CF (7 children/adolescents and 8 adults) on the combination treatment with Elexacaftor/Tezacaftor/Ivacaftor with 15 healthy controls, and they reviewed their BMD by doing a whole-body DXA scan. However, no differences were observed between the groups [34].

### 3.3. The Interplay Between CFTR Modulators and Muscle Mass and Strength in Children and Young Adolescents with CF

We identified seven studies that examined the impact of CFTR modulators on muscle mass and strength in children and adolescents with CF. One study was cross-sectional, three were observational, two were prospective, and one was multicenter. Their main characteristics are summarized in Table 2.

In addition to evaluating bone mineral density (BMD) in individuals with CF, Clayton LJ et al. also assessed peripheral muscle function and body composition in CF patients undergoing treatment with ETI, comparing these parameters to those observed in healthy control subjects [34]. Their findings did not reveal any differences between the groups in muscle strength, power, endurance, or body composition [34]. However, they documented a positive correlation between muscle strength and lean mass both in the study group and the control group [34].

Stallings VA et al. were the first to review the effect of ivacaftor in 2018 as a monotherapy in CF patients eligible for the treatment, including children above 5 years old [35]. Three months after the initiation of treatment, improvements were observed in both fat-free mass (0.9 ± 1.9 kg) and fat mass (1.6 ± 1.5 kg) [35]. Regarding muscle strength, improvements were also observed, including enhanced hand grip strength, increased jump height, greater peak power during vertical jump, and improved knee flexion strength [35].

Boat T et al. also evaluated the rates of change in body mass and muscle strength among children with cystic fibrosis (CF), aged 6 to 11 years, following initiation of ETI therapy [36]. These outcomes were compared to those observed in age-matched healthy controls [36]. They concluded that during Elexacaftor/Tezacaftor/Ivacaftor treatment, healthy and well-nourished children with cystic fibrosis demonstrated gains in muscle mass, although increases in fat mass often exceeded those in fat-free mass [36].

Imrei M et al. evaluated the effect of 24-month lumacaftor/ivacaftor (LUM/IVA) treatment in pediatric CF patients (median age: 9.3 years (5.5–14.2)) and they documented improvement of the Median BMI z-score from −0.81 (−1.37–0.49) to −0.39 (−0.88 to −0.04) (*p* = 0.288) [37]. Nevertheless, they suggested that the improvement in weight was driven entirely by fat gain and not muscle [37].

Anne-Sophie A et al. carried out a prospective study to assess the effects of tezacaftor and ivacaftor on muscle strength by measuring the quadriceps strength; however, no improvement was recorded following the treatment [38].

A study by Rysgaard UK et al. followed up patients with CF above 12 years old who were eligible for treatment with LUM/IVA and Tezacaftor/Ivacaftor therapy (TEZ/IVA) [39]. In the subgroup of patients who underwent assessments of muscle strength and muscle power, a statistically significant improvement was observed in both parameters at 6- and 12-month follow-ups (<0.001) [39]. In terms of body composition, fat mass increased significantly after twelve months of treatment, while lean mass remained stable [39].

Finally, a descriptive, observational, cross-sectional study by García-Pérez-de-Sevilla G et al. assessed and investigated the respiratory muscle function in CF children and adolescents under ETI in comparison with healthy individuals [40]. Specifically, when they measured the maximal expiratory and inspiratory pressures (MIP and MEP) and compared the results, they observed no significant differences despite the CF group presenting with less favorable lifestyle parameters [40]. Interestingly, they also reported that the MIP and MEP values in the CF group were not only comparable to those of healthy controls but also exceeded those previously reported in pre-ETI studies of children and adolescents with comparable FEV1 scores [40].

## 4. Discussion

In this narrative review study, we tried to evaluate the potential impact of CFTR modulators on bone density, muscle mass, and strength in children and young adolescents with CF. The administration of these new medications appears to exert a predominantly positive influence on skeletal muscle mass and strength, while concurrently impacting bone mineral density. While the available data remains limited, our analysis revealed interesting findings for clinicians who are involved in the care of patients with CF.

Improvements in the bone mineral density were mainly observed in the adult population, whereas similar results were not observed in the pediatric population. The sole study by Boni A et al., which included 24 children (8.7 ± 1.9 years), investigated only the baseline bone mineral density to report any differences among the different genetic types of cystic fibrosis [33]. The purpose of our review was not to compare the age groups, but to investigate whether CFTR modulators can affect the bone health of children and young adolescents with CF. However, most of the related studies included both children and adults in their study group. This variation in the results between the two age groups could be attributed to differences in skeletal maturation and hormonal influences. During puberty, sex steroids contribute significantly to bone turnover and skeletal maturation [41]. The epiphyseal fusion during bone maturation marks the transition to the remodeling process of bone metabolism [42]; therefore, CFTR modulators could provide more favorable results for bone mineral density in adulthood. Factors that contribute to the development of CF-related bone disease include vitamin D and vitamin K deficiency, malnutrition, calcium deficiency, delayed puberty and hypogonadism, reduced physical activity, respiratory infections and systemic inflammation, glucocorticoids, and CFTR dysfunction [17]. It is already recorded that CFTR mutations affecting chloride channel functionality or its potentiality to interplay with other proteins, might have impact on various signalling pathways such as Wnt/β-catenin, Nuclear Factor kappa-light-chain-enhancer of activated B cells (NF-κB), Mitogen-Activated Protein Kinase (MAPK)/Extracellular Signal-Regulated Kinase (ERK), or Transforming Growth Factor-beta (TGF-β), which are related to bone metabolism, whilst in CF, augmented NF-κB signaling, favors inflammation via monocytes and macrophages, which are both precursors of osteoclasts, following disruption of osteoblast differentiation and NF-κB prevents Wnt signaling, also leading to reduced bone formation and augmented bone resorption [43]. A study on homozygous F508del-CFTR mice reinforced the hypothesis that the F508del-CFTR mutation may contribute to bone disease by delaying new bone formation in infants and young children with cystic fibrosis, suggesting that CFTR-targeted treatments could be a valuable tool in the bone health of CF patients [21]. In the coming years, further research investigating the effects of CFTR modulators on bone health in the pediatric population with CF is warranted, as it may provide valuable insights into the long-term skeletal outcomes associated with these therapies.

In this review, we also addressed the effect of CFTR modulators on muscle mass and strength. Two studies, the first by Stallings VA et al. and the second by Rysgaard UK et al. showed that muscle strength improved at the 3-month and 6 to 12-month follow-up, respectively [35,39]. Besides hand grip strength, Stallings VA et al. also examined jump height, vertical jump, and knee flexion strength [35]. On the other hand, Anne-Sophie A. et al. and Clayton LJ et al. did not report similar results in their studies [34,38]. However, Clayton LJ et al. reported a positive correlation between muscle mass and strength, reinforcing the clinical relevance of lean tissue maintenance [34]. As for García-Pérez-de-Sevilla G et al., they investigated the effect of ETI on respiratory muscle strength and demonstrated inspiratory and expiratory pressures (MIP, MEP) comparable to healthy peers [40]. These results may suggest that ventilatory improvements noted in CF patients treated with ETI may be attributed not only to better airway clearance but also to enhanced respiratory muscle capacity. Notably, while our findings demonstrated beneficial effects of CFTR modulators on muscle mass, a few studies also reported a significant increase in fat mass, as depicted in Table 2. According to Imrei M et al., this phenomenon was observed particularly in the youngest age group, suggesting that initiating modulator therapy at an earlier age may enhance the recovery of pancreatic exocrine function [37]. As per Stallings VA et al. this trend could be attributed to decreased resting energy expenditure, increased dietary fat consumption, improved fat absorption, and reduced intestinal inflammation following the initiation of treatment with CFTR modulators [35]. The increase in fat mass reported in the above studies warrants the importance of thorough monitoring not only of weight but also of body composition as part of the regular screening of patients with CF while on treatment with CFTR modulators. According to the most recent ESPEN-ESPGHAN-ECFS guidelines, it is recommended to assess the fat mass and fat-free mass rather than sole reliance on BMI through the routine DXA scan for the monitoring of bone density [44]. It is also recommended that regular assessment of hand grip strength in individuals with CF aged 6 years and older may provide an early indication of muscle function decline and serve as a useful marker of nutritional status [44]. Additional guidance interventions, such as physical exercise and nutrition, should be offered.

This review has certain limitations. To begin with, the number of studies included was limited. There was also significant heterogeneity regarding sample size, type of CFTR modulator treatment, age ranges, and outcome measures. It should also be highlighted that while our main purpose was to study children and adolescent populations, many included studies mixed adult and pediatric cohorts. Moreover, the follow-up periods for some studies were too short; therefore, a clear need for long-term studies emerges so that the impact of CFTR modulators on bone and muscle health is better understood. Furthermore, the absence of randomization and appropriately matched control groups in many studies constrains the ability to draw robust causal inferences.

## 5. Conclusions and Future Perspectives

The current evidence of this review study supports that CFTR modulators may provide a beneficial impact beyond respiratory outcomes to bone health, muscle mass, and muscle strength in children and adolescents with cystic fibrosis. Enhancements in muscle mass and strength have been reported, particularly following treatment with ivacaftor as a monotherapy or ivacaftor as part of the triple therapy with elexacaftor/tezacaftor/ivacacaftor. The positive effect of CFTR modulators on bone health was mainly documented in the adult population. Our findings are promising; however, they highlight the need for further stratified studies.

## Figures and Tables

**Table 1:** Studies demonstrating the impact of CFTR modulators on bone density in children and young adolescents living with CF.

Authors/Ref	Study Design	Study Population	Main Results
Putman MS et al./[31]	Observational Study	26 CF subjects (15 adults/11 children) on ivacaftor 26 CF subjects not on ivacaftor 26 healthy volunteers	No differences in aBMD among the cohorts
Gur M et al./[32]	Pilot Study	9 adult CF subjects on ETI 9 CF controls not treated with ETI	ETI group: hip and spine BMD increased Control group: stable BMD
Boni A et al./[33]	Prospective study	24 children on ETI	No differences in baseline BMD among genetic groups
Clayton LJ et al./[34]	Cross-Sectional Study	15 CF subjects on ETI (7 children/adolescents 8 adults) 15 healthy controls	No important differences in BMD between the groups

Abbreviations: CF: Cystic Fibrosis; CFTR: Cystic Fibrosis Transmembrane; BMI: Body Mass Index; BMD: Bone Mineral Density; aBMD: areal Bone Mineral Density; ETI: Elexacaftor/Tezacaftor/Ivacaftor.

**Table 2:** Studies indicating the interplay between CFTR modulators and muscle mass and strength in children and young adolescents living with CF.

Authors/Ref	Study Design	Study Population	Main Results
Clayton LJ et al./[34]	Cross-Sectional Study	15 CF subjects on ETI (7 children/adolescents 8 adults) 15 healthy controls	No differences between the groups in muscle strength and body composition
Stallings VA et al./[35]	Multicenter Study	23 CF subjects 5–61 years of age on ivacaftor	Improved fat-free mass and muscle strength
Boat et al./[36]	Prospective Study	27 CF subjects 6–11 years of age on ETI 27 healthy controls	Increased muscle mass and fat mass
Imrei M et al./[37]	Observational Study	49 CF subjects 5.5–14.2 years of age on LUM/IVA	No change in muscle mass Increased fat mass
Anne-Sophie A. et al./[38]	Prospective Study	54 CF subjects 12 children/42 adults on tezacaftor and ivacaftor	No change in muscle strength
Rysgaard UK et al./[39]	Observational Study	91 CF subjects 21 children/70 adults on LUM/IVA and TEZ/IVA treatment.	Stable lean mass Improved muscle strength
García-Pérez-de-Sevilla G et al./[40]	Observational, cross-sectional study	24 CF subjects children and adolescents 6–18 years of age on ETI 24 healthy controls	Respiratory muscle strength comparable to that of healthy controls

Abbreviations: CF: Cystic Fibrosis; PI: pancreatic insufficiency; ETI: elexacaftor/tezacaftor/ivacaftor; DXA: Dual-energy X-ray absorptiometry; LUM/IVA: lumacaftor/ivacaftor; BMI: Body Mass Index; ELX/TEZ/IVA: elexacaftor-tezacaftor-ivacaftor; TEZ/IVA: Tezacaftor/Ivacaftor.

## Data Availability

No new data were created or analyzed in this study.
